# Rare Causes of Hypoglycemia—Lessons from Case Reports

**DOI:** 10.1210/jcemcr/luaf270

**Published:** 2025-11-20

**Authors:** Alia Munir

**Affiliations:** B Floor Endocrine Unit, Royal Hallamshire Hospital, Sheffield, South Yorkshire S11 9RQ, UK

**Keywords:** hypoglycemia, insulinoma, Whipple triad, congenital hyperinsulinism, continuous glucose monitoring, multidisciplinary team

## Introduction

Hypoglycemia in individuals without exogenous insulin use is uncommon and heterogeneous, with an estimated frequency of 36 per 10 000 hospital admissions [1]. Serious sequalae may occur, particularly with missed or delayed diagnosis. Although rare, here we reflect on 3 recently published case reports on the clinical theme of nondiabetic hypoglycemia and review the unique aspects of the presentation, diagnosis, and treatment. The mechanisms contributing to hypoglycemia in the 3 case reports differ, ranging from a tiny β-cell neuroendocrine tumor secreting insulin autonomously [2], dysautonomia in Ehlers-Danlos syndrome (EDS) with gastrointestinal (GI) dysmotility and amplify postprandial insulin excursions [3], to a novel heterozygous mutation in the glucokinase (*GCK*) gene, lowering the glucose threshold for insulin secretion [4]. Together, these reports remind us to first use a mechanism-based approach to diagnosis. Returning to first principles of a detailed medical history followed by a clinical review of the patient to ensure fulfillment of Whipple triad is key. Then, it is important to classify the hypoglycemia in relation to timing of food intake and tailor subsequent investigations in line with Endocrine Society [5] and European Neuroendocrine Tumor Society (ENETS) guidance [6]. The aim should be timely diagnosis with avoidance of overmedicalization and investigation with early multidisciplinary collaboration.

## Lesson 1: Use of receptor-targeted imaging and multidisciplinary team-working

The work by Arjunan et al, *In the Hunt for an Insulinoma* [2], the most common of the functional pancreatic neuroendocrine tumors, demonstrated fulfillment of the Whipple triad and unequivocal biochemistry using the gold-standard test of the supervised fast [5]. This was a truly multidisciplinary, internationally coordinated case with the patient care being coordinated and delivered in Dubai, India, and London. Having confirmed endogenous hyperinsulinemic hypoglycemia—elevated insulin, C-peptide, and proinsulin levels and with suppressed β-hydroxybutyrate and a negative sulfonylurea screen—standard localization techniques, including the use of ultrasound scan (USS) of the abdomen, magnetic resonance cholangiopancreatography, and 3T magnetic resonance imaging (MRI) failed to locate a lesion. Furthermore, endoscopic ultrasound scan (EUS) and gallium 68 (^68^Ga) 1,4,7,10-tetraazacyclododecane-1,4,7,10-tetraacetic acid (DOTA)-octreotate (DOTATATE) gallium 68 (^68^Ga) 1,4,7,10-tetraazacyclododecane-1,4,7,10-tetraacetic acid (DOTA)-octreotate (DOTATATE) ^68^Ga-Dotatate positron emission tomography–computed tomography (PET-CT) were also unhelpful. Even though repeat EUS showed a possible 4-mm lesion, cytology was negative.

The authors describe the use of receptor-targeted imaging using ^68^Ga-exendin-4 PET-CT to locate the lesion confidently in the uncinate process, with an overall accuracy of 94.6%. This imaging technique uses the expression of glucagon-like peptide-1 receptors on the tumor surface. Further confirmation with intraoperative USS allowed for curative surgery through enucleation, preserving as much pancreas as possible. The availability of this tracer is limited, and collaboration between centers is essential. There is a growing body of evidence prioritizing this imaging hierarchy and use of advanced tracer biology when conventional imaging fails [7]. Selective arterial calcium stimulation (SACST) remains a valuable test to regionalize the territory of insulin secretion when used in expert hands. Continuous glucose monitoring (CGM) was used as an adjunct to management of hypoglycemia before and to review glycemia after definitive treatment.

Practical implications here include the following:

- Whipple triad with the “critical samples”
- Multidisciplinary team management and international coordination of care
- Where imaging is negative, refer for ^68^Ga-exendin-4 PET-CT or consider SACST within an expert center.

## Lesson 2: Ehlers-Danlos syndrome is underdiagnosed; symptoms may overlap and require careful evaluation

Saeed and colleagues [3] describe a woman with hypermobile EDS (hEDS) and predominantly reactive or postprandial hypoglycemia accompanied by autonomic symptoms, joint mobility, and GI complaints. The authors provide a useful algorithm for work-up of potential hypoglycemia and to ensure alternative causes are excluded, including adrenal insufficiency. In the reported case, 3 separate overnight fasts and an observed fast were used to exclude hyperinsulinism and confirm appropriate suppression of insulin and ketogenesis when hypoglycemic. They highlight the challenges posed by hEDS, such as diagnostic mimicry and overlap. It is pertinent to note the initial assessment in the case report was for seizure-like activity. Mechanistically, hEDS coexists with dysautonomia and rapid GI emptying, accelerating carbohydrate delivery and exaggerating secreted insulin excursions modulating reactive or postprandial hypoglycemia. The authors note there are several mechanisms influencing hypoglycemia from the EDS-related autonomic dysfunction effects on Glut2-dependent glucose sensing neurons, gluconeogenesis and vagal hypertonia.

Saeed et al [3] eloquently outline the key to management being nutrition led. Patient education under the guidance of a multidisciplinary team, which should include an experienced dietitian who can provide advice on frequent, small, and low-glycemic index meals, is key. The effect of exercise is also noted.

The practice points here include the following:

- To validate true hypoglycemia
- Nutrition-led management
- CGM helped map triggers with the emphasis on venous/capillary confirmation of hypoglycemia during symptoms remaining essential.

## Lesson 3: Congenital hyperinsulinism may present later and be milder

Here, the authors [4] report a rare hereditary disease affecting a mother and daughter with mild congenital hyperinsulinism (CHI) due to a novel activating *GCK* variant (*c.*212T > C;p.Val71Ala) and a second variant of unknown significance, *ABCC8*. Biochemistry was consistent with insulinoma but imaging, including EUS, CT, and MRI, failed to localize a lesion. The authors noted asymptomatic hypoglycemia after the ingestion of fast-acting carbohydrate. They also noted the mother's asymptomatic hypoglycemia, and this instigated the genetic testing. Persistent hypoglycemia in neonates and infants is most frequently caused by CHI with 16 implicated genes. The phenotype reflects a lowered set point for insulin release, rather than tumoral secretion, with mild hypoglycemia managed with diet and glucometer usage. Diazoxide was reserved for the development of persistent symptomatic hypoglycemia. Recognition of this mechanism is important when endogenous hyperinsulinism is proven, noting the age of onset, duration of symptoms in the absence of other endocrinopathies, and negative imaging in the context of a family history of hypoglycemia. Here, genetic confirmation guides expectation, family counseling, and avoids unnecessary use of time and resources.

Practice points include the following:

- Consider genetic testing in persistent hypoglycemia, suggestive family history, and negative imaging
- A genetic diagnosis allows for informed family counseling and tailored therapy

Some forms of glucose monitoring were used in all cases; the first two used CGM and the last case capillary blood glucose monitoring. CGM appears to be a useful adjunct, giving insights into glucose dynamics: timing, patterns, and treatment response. Saeed et al [3] demonstrated its use in the hEDS case, identifying timing and severity of episodes and guiding dietary modifications. It is important to that note sensor accuracy declines at very low values and should not be used alone for diagnosis or to establish etiology. One limitation of CGM is that interstitial glucose levels may lag behind the plasma glucose during rapid changes. Nevertheless, CGM does enhance clinical management and patient empowerment [8].

Across the case vignettes, the key recurring lessons emphasize the need for careful clinical evaluation and work-up, early referral to expert centers, multidisciplinary team discussion, and coordinated management. Being mindful of overmedicalization, symptom dismissal, access and availability of newer imaging modalities, and the role of genetics in selected patients with proven endogenous hyperinsulinism will help reduce morbidity and time to diagnosis with a global aim for precision and high-quality care in endocrinology.

## Abbreviations

^68^Ga: gallium 68
CGM: continuous glucose monitoring
CHI: congenital hyperinsulinism
EDS: Ehlers-Danlos syndrome
EUS: endoscopic ultrasound scan
*GCK*: glucokinase gene
GI: gastrointestinal
hEDS: hypermobile Ehlers-Danlos syndrome
MRI: magnetic resonance imaging
PET-CT: positron emission tomography–computed tomography
SACST: selective arterial calcium stimulation
USS: ultrasound scan

## References

[1] Nirantharakumar K, Marshall T, Hodson J, et al Hypoglycemia in non-diabetic in-patients: clinical or criminal? PLoS One. 2012;7(7):e40384. 22768352 10.1371/journal.pone.0040384 PMC3388042

[2] Arjunan D, Grossman AB, Singh H, Rai R, Bal A, Dutta P. A long way to find a small tumor: the hunt for an insulinoma. JCEM Case Rep. 2024;2(11):luae192. 39465233 10.1210/jcemcr/luae192 PMC11505447

[3] Saeed H, Sheehan A, Patti ME. Hypoglycemia associated with hypermobile Ehlers-Danlos syndrome. JCEM Case Rep. 2024;2(11):luae205. 39498469 10.1210/jcemcr/luae205 PMC11532648

[4] Sozaeva LS, Ismailova SK, Chernyak IY, Popov SV, Zakharova VV, Chugunov IS. Mild congenital hyperinsulinism caused by mutation in human *Glucokinase* gene. JCEM Case Rep. 2024;2(12):luae226. 39659388 10.1210/jcemcr/luae226 PMC11630036

[5] Cryer PE, Axelrod L, Grossman AB, et al Evaluation and management of adult hypoglycemic disorders: an Endocrine Society Clinical Practice Guideline. J Clin Endocrinol Metab. 2009;94(3):709‐728. 19088155 10.1210/jc.2008-1410

[6] Hofland J, Falconi M, Christ E, et al European Neuroendocrine Tumor Society 2023 guidance paper for functioning pancreatic neuroendocrine tumour syndromes. J Neuroendocrinol. 2023;35(8):e13318. 37578384 10.1111/jne.13318

[7] Boss M, Eriksson O, Mikkola K, et al Improved localization of insulinomas using (68)Ga-NODAGA-exendin-4 PET/CT. J Nucl Med. 2024;65(12):1959‐1964. 39419553 10.2967/jnumed.124.268158 PMC11619583

[8] Worth C, Worthington S, Auckburally S, et al First accuracy and user-experience evaluation of new continuous glucose monitoring system for hypoglycemia due to hyperinsulinism. J Diabetes Sci Technol. 2024;19(5):1280‐1285. 38616550 10.1177/19322968241245923 PMC11572253

